# Predictors of Children’s Early Numeracy: Environmental Variables, Intergenerational Pathways, and Children’s Cognitive, Linguistic, and Non-symbolic Number Skills

**DOI:** 10.3389/fpsyg.2020.505065

**Published:** 2020-11-06

**Authors:** Luca Bernabini, Valentina Tobia, Annalisa Guarini, Paola Bonifacci

**Affiliations:** ^1^Department of Psychology, University of Bologna, Bologna, Italy; ^2^Faculty of Psychology, Vita-Salute San Raffaele University, Milan, Italy

**Keywords:** early numeracy, broader phenotype, approximate number system, parents, math skills

## Abstract

Early numeracy skills in preschool years have been found to be related to a variety of different factors, including Approximate Number System (ANS) skills, children’s cognitive and linguistic skills, and environmental variables such as home numeracy activities. The present study aimed to analyze the differential role of environmental variables, intergenerational patterns, children’s cognitive and linguistic skills, and their ANS in supporting early math skills. The sample included 64 children in their last year of kindergarten and one parent of each child. Children were administered a battery of cognitive and linguistic tasks, and a non-symbolic comparison task as a measure of ANS. Parents were administered similar tasks assessing cognitive skills, math skills, and ANS skills (estimation and non-symbolic comparison), together with a questionnaire on home numeracy. Results showed that home numeracy predicted children’s early math skills better than a number of parent and child variables. Considering children’s skills, their ability in the non-symbolic magnitude comparison task was the strongest predictor of early math skills. Results reinforce the importance of the role of home numeracy activities and children’s ANS skills above that of parents’ math skills.

## Introduction

Early numerical abilities, those that involve the understanding of magnitudes and the development of numerical processing skills, are manifested during the first few months of life in humans from various cultural backgrounds (Simon et al., 1995; Gordon, 2004; Xu et al., 2005). However, many different factors contribute to the development of mature math skills and the turning point between the end of preschool and the beginning of primary school resulted to be an important moment in which to understand how the spontaneous and intuitive basic calculation skills develop (Jordan et al., 2009).

From a theoretical viewpoint, the neuroconstructivism framework (Westermann et al., 2007) suggests that, when trying to explain developmental pathways of cognitive skills, different levels of analysis should be taken into account, including biological, cognitive, behavioral, and environmental components, as well as their reciprocal relationships. Considering the development of specific learning skills, the multiple deficit model (Pennington, 2006) evidenced the need to overcome the analysis of single markers in favor of multiple indexes, and increasing attention has been dedicated to intergenerational models (van Bergen et al., 2015) which extends to parents’ skills and family characteristics the set of variables to be considered in analyzing the development of learning skills. However, regarding intergenerational models, most studies were referred to literacy skills (Bonifacci et al., 2014; van Bergen et al., 2015) and very few studies tried to apply this model to the development of math skills (Braham and Libertus, 2017; Navarro et al., 2018) but these studies did not account for variables related to children’s cognitive and linguistic skills.

In the present study, focused on children at the end of preschool years, we took account of many of the candidate factors that previous literature suggested to be related to math skills development, including (1) environmental variables, (2) parents’ math skills and (3) children’s cognitive and early math skills. In the following sections, in order to better account for the selection of the candidate predictors included in the study, we will briefly revise previous evidence for these three domains.

### Environmental Predictors of Math Skills

Differences in the quality and quantity of children’s early math learning opportunities have been shown to be related to their consequent math performance (Hill et al., 2005; Levine et al., 2010; Maloney et al., 2015; Tobia et al., 2016). Indeed, many pieces of evidence now indicate that parents matter in the development of children’s math skills, and recognize the influential role of home numeracy activities (LeFevre et al., 2009), defined as the parent-child interactions that include experiences with numerical content in daily-life settings (Mutaf Yildiz et al., 2018b). Previous research found evidence of the usefulness of home literacy, that is, exposure to books and reading in the familiar context, in the expansion of vocabulary and decoding skills (Sénéchal and LeFevre, 2001). Similar evidence has been found regarding the role of home numeracy activities in the development of math and arithmetic skills (Kleemans et al., 2012). Home numeracy, indeed, can be conceived as a multifaceted domain, and its relationship with children’s numeracy skills might be differentiated on the basis of direct versus indirect activities (LeFevre et al., 2009; Skwarchuk et al., 2014). The first focus on counting and teaching numbers and have been found to be related to the development of children’s symbolic abilities, whereas the second involve playing games with numbers (e.g., dice), or doing household activities where you need to count, and have been found to be related to children’s non-symbolic abilities. Other authors also highlighted the importance of “math talk,” that is, how parents use math or number words in everyday life (Braham et al., 2018). In this regard, Elliott et al. (2017) found that parents’ use of numbers larger than 10 was positively and significantly related to children’s math abilities even when controlling for parents’ overall talk. It has also been found that intervention directed to parents leads to enhanced home numeracy activities and significant gains in children’s early numerical skills (Niklas and Schneider, 2014). However, a few studies found a non-significant association between home numeracy and children’s performance in early math (e.g., Blevins-Knabe et al., 2000), and some other studies showed that there were differential effects of formal and informal home numeracy activities on different domains of number processing skills (Manolitsis et al., 2013; Kleemans et al., 2016; Mutaf Yildiz et al., 2018a). For example, Mutaf Yildiz et al. (2018a) found that formal home numeracy was related to enumeration skills, informal home numeracy was related to calculation and symbolic processing, but there were no relationships with non-symbolic processing.

In sum, although most researchers agree that an enriched home numeracy environment is positively related to early numeracy skills in children, the precise causal patterns are far from clear, as cognitive and linguistic factors impact numerical abilities as well. A possible limitation of previous studies addressing the direct link between home numeracy and children’s numeracy skills was that a limited number of possible intervening variables that might reduce or hamper the strength of the relationship was considered (Carroll et al., 2019).

Finally, within a multilevel perspective, research examining the impact of family-related factors in children’s cognitive skills, should include the measurement of socio-economic status (SES) (Bradley and Corwyn, 2002). Although the relationships between academic achievement and SES was not consistent across studies (Sirin, 2005), both home numeracy activities and children’s early math skills might be related to SES and therefore the role of SES should be included. Although there is relatively little research on the specific relationship between SES and math skills, most authors suggested a positive relationship (Duncan and Magnuson, 2011; Reardon and Portilla, 2016), although with significant differences between countries and different school systems (Baird, 2012). Furthermore, SES disparities have been found to be differently related to subcomponents of math skills, with higher gaps in the verbal aspects of maths skills, and minor or no differences in the performance in non-verbal and non-symbolic tasks (Jordan et al., 1992; Jordan and Levine, 2009). There was, also, contrasting evidence concerning the relationship between SES and the quantity of home learning activities. Silinskas et al. (2010) showed that the lower the SES of mothers and fathers, the more teaching of reading and mathematics they reported; also, the lower the children’s academic performance at the beginning of primary school, the more teaching by mothers and fathers was reported. These results showed that parents adaptively adjusted their teaching to the child’s academic performance level, even when they had low SES. Similar results were found by LeFevre et al. (2010) and Niklas and Schneider (2014). It is, however, possible that higher SES children were exposed to higher quality numeracy activities compared to lower SES children (Elliott and Bachman, 2018).

### Intergenerational Paths of Math Skills

In recent years, increasing research has focused on intergenerational transmission of cognitive skills in parents and children. The first line of research regarded the concept of a broader phenotype of developmental disorders, which refers specifically to the cognitive endophenotypes that are shared with unaffected family members. Endophenotypes are heritable biochemical, endocrinological, neuroanatomical, or neuropsychological constituents of disorders, although they are likely to be influenced by complex interactions between genes and environments (Caspi and Moffitt, 2006). Studies conducted on children with dyslexia revealed, for example, that phonological deficits are shared in unaffected family members, and that parents and siblings of children with dyslexia underperform in reading measures compared to family members who are not at risk for the disorder (Göbel and Snowling, 2010). Bonifacci et al. (2014) found that parents of children with dyslexia underperformed in phonological and decoding tasks compared to parents of typical readers, with significant relationships between parents’ and children’s reading skills. As far as math is concerned, Shalev et al. (2001) suggested that dyscalculia, i.e., a specific learning disorder affecting mathematics (American Psychiatric Association, and Taskforce on Dsm-5, 2013), was a “familiar disorder,” with higher percentages of family members of children with dyscalculia showing impaired performances in math tasks, compared to the general population. In a similar vein, Desoete et al. (2013) found that 33% of siblings of children with dyscalculia had clinical or subclinical scores in early arithmetic skills and were at risk of developing dyscalculia: this percentage is above expectations based on typically-developing children. Therefore dyscalculia, like other specific learning disorders, was characterized by a significant family aggregation, suggesting a role of genetics in the evolution of this disorder (Shalev et al., 2001). However, twin studies found contrasting results on the genetic determinants of math ability. Some studies reported expected heritability for low mathematical performance ranging from 0.65 (Haworth et al., 2009) to 0.69 (Oliver et al., 2004); others proposed that basic numerical understanding was only moderately heritable, with environmental influences being a more powerful predictor (Tosto et al., 2014). Two genome-wide association studies failed to find any proper association (Docherty et al., 2010; Baron-Cohen et al., 2014), while Davis et al. (2014) confirmed a significant genetic component underlying mathematical abilities. Finally, Ludwig et al. (2013) found that the rs133885 variant in the myosin-18B (MYO18B) gene was associated with mathematical ability at a statistically significant level, but this result was not replicated by Pettigrew et al. (2015).

In summary, although previous evidence suggests a plausible genetic component in math intergeneration skills, the debate is still open and the role of environmental variables seems to be higher compared to studies on reading skills. Although very few studies have specifically assessed parents’ math skills, it might be that parents’ math skills may affect their math-related interactions with children at home. Parents’ with stronger math skills may be more interested in math and, for example, engage their children in math activities more often or, when possible, select a preschool with a more math-focused curriculum.

Recently, some studies addressed the issue of intergenerational transmission of literacy (van Bergen et al., 2015) and math (Braham and Libertus, 2017; Navarro et al., 2018; Bernabini et al., 2020) skills not only in children with dyscalculia but also in typical populations. Navarro et al. (2018) found that parents’ Approximate Number System (ANS) skills were related to toddlers’ number processing and that this relation was independent of children’s vocabulary or parents’ perceived math ability, suggesting a specific intergenerational transmission of the ANS. In another study (Braham and Libertus, 2017), conducted on 54 children (5–9 years old) and their parents, children’s ANS acuity positively correlated with their parents’ ANS acuity. Also, children’s math abilities were predicted by unique combinations of parents’ ANS acuity and math ability depending on the specific math skill in question. Nevertheless, to our knowledge, there is a paucity of research that considers the differential role of home numeracy, parents’ skills, and children’s own skills in a comprehensive model.

### Children’s Cognitive, Linguistic and ANS Skills

The ability of humans (and animals) to process magnitudes even prior to the development of verbal skills is thought to be mediated by an intuitive, non-symbolic system defined as the ANS that includes the ability to quickly understand and manipulate numerical quantities (Dehaene, 1997). This precocious and preverbal sense of numerical magnitude seems to represent the basis on which, through the interplay with other cognitive skills (mainly language and working memory), numerical knowledge develops from preschool to primary school, when formal teaching shapes arithmetic ability (Xu and Spelke, 2000; Feigenson et al., 2004; Xu et al., 2005; Halberda and Feigenson, 2008; Izard et al., 2009; Nieder and Dehaene, 2009; Brannon and Merritt, 2011).

According to Von Aster and Shalev’s (2007), the development of number representation is hierarchically organized. In their four-step model, as the first step, core-system representation of cardinal magnitude and various functions, such as subitizing and approximating, implements the basic meaning of the number. This step is a necessary precondition for children to learn to connect a perceived number of objects or events with spoken or, later, written and Arabic symbols. As the second step, children learn the verbal number system and, as the third step, the Arabic symbolization. These two steps are, in turn, the precondition for the evolution of a mental number line (step 4) in which ordinality is represented as a second core aspect of number processing. The first step produces the foundation for the subsequent acquisition of all numerical skills. Children who do not show these primordial skills may be able to learn the names of the numbers but do not associate these names with the meaning of a quantity. From the first to the fourth step, the model predicts a progressive increase of working memory load in numerical processing.

In typically-developing children, several studies have revealed a strict relationship between dot comparison – as a measure of ANS - and mathematics achievement (Libertus et al., 2011; Mazzocco et al., 2011). However, there are contrasting results to this regard (for a review, see De Smedt et al., 2013), and some authors suggest that symbolic representation is more strictly related to mathematical achievements (Göbel et al., 2014; Goffin and Ansari, 2019). This is in line with the strong relationship found between math skills and language development (e.g., Korpipää et al., 2019), supporting the idea that language competence may act as a scaffolding ability on which numerical development may rely (Bonifacci et al., 2016). In terms of linguistic skills, the verbal code proposed within the Triple Code Model (Dehaene, 1992, 1997) was used in particular for counting, addition, and easy multiplication. From a developmental perspective, and in line with Von Aster and Shalev’s (2007) model (step 2), the verbal code allowed children to associate symbols to quantities. Therefore, language was essential for the growth of numerical competencies. In particular, lexical amplitude (vocabulary) was necessary to understand specific math terms (Adams, 2003; Purpura et al., 2011), and phonological awareness might play a role in the storing and retrieval of numbers (Swanson and Sachse-Lee, 2001).

Furthermore, many studies have reported relationships between early numeracy skills and other cognitive skills. Executive functions (EFs), defined as cognitive processes which serve for selecting and successfully monitoring goal-directed behaviors, have been found to be related to early (Espy et al., 2004; Purpura et al., 2017; Schmitt et al., 2017) and late development of math skills (Cragg et al., 2017). Actually, some studies suggested that EFs, and in particular inhibitory control, might drive the relationship between ANS acuity and math ability in young children (Fuhs and McNeil, 2013) or, even, that the association between ANS and math skills might be better explained by the inhibitory control demands of some trials of the dot comparison task (Gilmore et al., 2013; Leibovich and Ansari, 2016). Finally, visuo-spatial working memory, that is, the ability to retain information for a short time in order to think about it, has been documented to be highly related to non-symbolic and written arithmetic and word problems (Zhang and Lin, 2015), counting (Cirino, 2011), and magnitude judgments (Simmons et al., 2012).

In summary, the different potential factors that might be related to math skills, above reviewed within the three main domains of analysis, strongly suggest that the emergence of formal mathematical competencies results from the complex interplay between the ANS and other cognitive skills and is further related to environmental factors and intergenerational paths. Despite many investigations addressed each of these factors separately, few studies attempted to examine multi-domains relationships with children’s math skills.

### The Current Study

The present study aimed to investigate which components are related to early math skills in preschool children, within a multilevel theoretical framework that accounts for intergenerational paths, demographic and environmental factors and children’s cognitive and linguistic skills. In particular, our objective was to explore the role of SES and environmental stimuli (home numeracy), intergenerational patterns (parent’s math skills), children’s cognitive and linguistic skills, and children’s ANS in supporting children’s early math skills. To do so, a battery of tasks assessing prerequisites of math and ANS skills was administered to a sample of children during their last year of kindergarten, taking into account cognitive measures such as attention, visuo-spatial memory, and non-verbal IQ, as well as language skills. Another battery of tasks assessing math abilities, including ANS measures, was administered to parents.

(1) As a first step, we analyzed the correlations of children’s early math skills with the following variables included in the study:
  a. Familiar SES and home numeracy (environmental variables). In line with past studies (e.g., LeFevre et al., 2009), positive associations between children’s early math skills and environmental variables were expected.
  b. Parents’ cognitive and mathematical basic (i.e., ANS) and more advanced (i.e., calculation) abilities (intergenerational transmission). Based on previous literature, we expected significant associations between children’s early numeracy skills and their parents’ ANS skills (see Navarro et al., 2018). Our analysis of the link with parents’ calculation skills is more explorative, but we expected to see at least a weak association with children’s early math.
  c. Children’s competence in the cognitive and linguistic domains (inter-domain children’s variables). We hypothesized significant links between early math skills and domain-general cognitive variables, and in particular with working memory, for which literature provides a body of evidence (Raghubar et al., 2010). Also, we expected to find an association with language variables, as shown by past studies on preschoolers (Bonifacci et al., 2016).
  d. Children’s competence in ANS skills (intra-domain children’s variables). We hypothesized significant links between early math skills and ANS, as shown in previous literature (Mazzocco et al., 2011).
(2) As a second step, in order to test which of the above relationships was more strongly related to early math skills, we investigated the concurrent predictors of children’s early math skills with a three-steps regression model on children’s composite numeracy score, that included: (1) the environmental variables and the intergenerational transmission represented by parents’ skills, (2) children’s inter-domain (cognitive and linguistic) skills, and (3) intra-domain (ANS) skills as potential predictors. The aim of this analysis was to better understand the strength of these multiple factors as concurrent predictors of children’s early math skills. In particular, the aim was to disentangle, within environmental and intergenerational variables, if the parents’ abilities in mathematical tasks, in particular the ANS tasks, were predictive of their children’s performances when considered together with home numeracy activities. Then, adding children’s skills, we wanted to evaluate if these explained additional variance and which skills, amongst inter-domain and intra-domain, predicted children’s math skills above and beyond environmental and parents’ variables.

We hypothesized that, in line with Braham and Libertus (2017) and with Navarro et al. (2018), parents’ ANS, as measured by non-symbolic comparison, would predict children’s ability with numbers. However, with a sample of 4–5-year-old children, we expected that the role of the home numeracy would also be significant, along with children’s ANS skills.

This is the first study that directly investigates the link between preschool children’s and parents’ math skills, including measures of ANS, basic symbolic skills, and more complex calculating skills. This research design contributes to disentange the intergenerational role of both basic non-symbolic numerical skills and home numeracy in predicting math ability in children, as measured by a battery of ecological and complex tasks.

## Materials and Methods

### Participants

The sample included 64 children (mean age = 5.72 years, SD = 0.53, range = 4.42–6.58; 45.3% females), attending their last year of kindergarten. For each child, a parent was involved in data collection. Most of the parents were mothers (87.5%; mean age = 40.53 years, SD = 4.64, range = 29–49); in the remaining cases fathers were involved (mean age = 45.14 years, SD = 8.84, range = 28–54). Mean Non-verbal IQ (standardized scores) was 103.2 (SD = 15.3) for children and 99.0 (SD = 14.5) for parents. Parents’ mean SES was in the medium-high interval (mean = 45.4, SD = 12.6), and 86,5% of parents had a medium to high SES (see section “Materials and Methods” for details on the instruments used). There were no significant differences (all *p*s > 0.1) between fathers’ and mothers’ educational level (fathers: mean = 4.83, SD = 0.98; mothers: mean = 5.4; SD = 1.22) and occupation (fathers: mean = 5.33, SD = 2.6; mothers: mean = 6.16 SD = 2.4). Parents who volunteered to participate in the study were tested by the first author of the study and they stated that they spent time with their children in everyday activities. All children and parents were native Italian speakers. None of the children included in the study had been referred for neurodevelopmental disorders.

Participants were selected from four public preschools in suburban areas in Northern Italy. Italian preschools do not provide formal instruction regarding reading, writing, or mathematical skills, although children are involved in pre-reading and pre-writing activities that aim to familiarize them with letters and letter-sound correspondence. From an initial sample of 69 children, in the study we included only participants with a complete dataset collected from the child and one of the parents. Parents provided written informed consent prior to the experiment. The study was approved by the Ethical Committee of University of Bologna.

### Materials

Parents and children were administered tests assessing intellectual functioning, formal math skills, and symbolic and non-symbolic comparison tasks. Parents were also administered a socio-demographic questionnaire and a questionnaire investigating home numeracy habits. A detailed description of the tasks is detailed below.

#### Socio-Demographic Information

The Hollingshead Four Factor Index of Social Status (Hollingshead, 2011) was used. For this study, indexes of educational level (EL), and occupation (O) were chosen. For both indexes, scores ranged from 1 to 9. SES scores for fathers and mothers were managed with the formula EL^∗^3 + O^∗^5, and an aggregate SES score for children resulted from the mean of the two values. For example, a parent with a high school degree would receive a score of 4 and employed as a carpenter corresponds to a score of 4; therefore, the total SES for this parent would be of 12 + 20, equals 32 (mean range). A parent with a Ph.D. and employed as engineer would receive, respectively, scores of 7 and 8 with a total SES of 21 + 40 = 61 (high range). Scores ranged from a minimum of 8 to a maximum of 66; suggested classification for interpreting the scores is given by the questionnaire’s instructions (8–19 low; 20–29 medium-low; 30–39 medium; 40–54 medium-high; 54–66 high; Hollingshead, 2011).

#### Children’s Assessment

##### Cognitive skills

###### Non-verbal IQ

Children were administered the *Matrices* subtest of K-BIT 2 (Kaufman and Kaufman, 2004; Bonifacci and Nori, 2016). This test measured Non-Verbal IQ; it had different starting points based on the participant’s age and stops after four consecutive wrong responses. For children aged 4–5 years the first nine items required to point to the image (amongst five images) that was associated with the target image (e.g., a car goes with a truck). Then, from item 10 children were required to solve simple analogies with concrete objects (e.g., The car goes with the road, the boat goes with …) and, increasing in difficulty, with abstract figures. Raw scores (the maximum score was 46) were converted into standard scores (see participants description) according to the Italian test manual (Bonifacci and Nori, 2016) and z-scores (for calculating the Composite Cognitive Score). Split-half reliability coefficient in developmental age (4–18 years) was 0.87.

###### Visual-spatial memory

Children were administered the visual-spatial memory task from the SNUP test (Tobia et al., 2018). In this task, children had to remember the positions of one to four elements on 3 × 3 and 4 × 4 grids that were presented for 2 and 4 s, respectively, and then covered. A total of 10 grids, preceded by an example, were presented, and a score of 1 was assigned for each element remembered in the correct position, for a maximum total score of 26 (Cronbach’s α = 0.80). Z-scores derived from the test manual were used in the analyses.

###### Attention

The visual attention task from the NEPSY-II (Korkman et al., 2014) was administered to the children in order to assess selective and sustained attention. The visual attention task is a visual cancelation task, which requires children to identify and mark the target stimulus (a rabbit) from among an array of distractors as quickly as possible. The variable considered was accuracy, measured as the difference between the total number of target stimuli identified and the incorrect targets marked (i.e., distractors). The maximum score is 27. Z-scores derived from the test manual were used in the analyses. The Cronbach’s α reliability for this measure was 0.76.

A Composite Cognitive Score was computed with mean z-scores of non-verbal IQ, visuo-spatial memory and attention tests.

##### Language skills

Children were administered two subtests of the IDA battery (Bonifacci et al., 2015) to assess vocabulary and phonological awareness. For all the subtests, z-scores derived from the test manual were used.

###### Vocabulary

Children were asked to name 36 images selected for their decreasing frequency in spoken language. The accuracy score, ranging from 0 to 36, was considered. The scale’s Cronbach’s alpha was 0.85.

###### Phonological awareness

To assess children’s phonological awareness, a syllable segmentation task was administered. Stimuli were presented orally, and children were required to provide a verbal answer by segmenting sounds (e.g., Carota ? Ca-ro- ta; Carrot; six items). Each item received a score of 1 for correct responses and a score of 0 for incorrect answers, for a maximum total score of 6. The scale’s Cronbach’s alpha was 0.84.

A Composite Language Score was computed by calculating mean z-scores for the Vocabulary and Phonological Awareness score.

##### Early numeracy

Children were administered four subtests of the SNUP test (Tobia et al., 2018) to assess the children’s early numerical skills. For all the subtests, z-scores derived from the test manual were used.

###### Counting and biunivocal correspondence

Children were asked to count 20 buttons scattered on a board measuring 20 cm × 30 cm. Knowledge of the verbal sequence of numbers and the acquisition of the biunivocal correspondence principle of counting, namely the ability to link each number word to an individual object, were evaluated separately. Scores range from 0 to 20 for each subscale, and one point was given for each number word named correctly on the scale of 1–20 and when the child linked one number word to one button. The scale’s Cronbach’s alpha was 0.93.

###### Recognition and reading of digits

Semantic knowledge of digits, that is, recognition and reading, were assessed for digits 1 to 9. The task was organized as a game with numbers comparable to bingo. A card containing the digits from 1 to 9 randomly allocated on a grid amongst blank squares was used, together with a small bag containing nine number cards, each representing a digit. In the digit recognition subtask, children pointed to the number on the bingo card that had been picked out of the bag and read aloud by the examiner. For the digit reading subtask, children picked a number from the bag and read it aloud. For each digit correctly identified or read, a score of 1 was given (total score: 0–9 for each subtask). The subtest’s Cronbach’s alpha was 0.93.

A Composite Numeracy Score was computed by calculating mean z-scores for the counting, biunivocal correspondence, and recognition and reading of digit scores.

##### Speed of processing and ANS

###### Speed of processing

A task to measure simple reaction times (RTs, Bonifacci and Snowling, 2008) was administered. Children were required to press the space bar of the keyboard, as fast as they could, whenever a ‘blue star’ (measuring 8 cm × 8 cm) appeared on a white screen. The target stimulus was presented on the screen for a maximum of 1 s and disappeared after the response was made. The following stimuli appeared at 1-s intervals after the preceding stimulus had disappeared. Fifteen practice trials were completed, followed by 40 test trials. Mean RTs were recorded.

###### ANS – non-symbolic magnitude comparison

A computerized magnitude comparison task was administered. Children were presented with two sets of dots in a random configuration, and were asked to identify the set representing the larger numerosity, by pressing one of two keys on a computer keyboard (W and P); they were instructed not to count. After a practice block (10 trials), 64 randomized trials were administered, with pairs of stimuli ranging from 1 to 9 dots and numerical distance between them ranging from 1 to 8. Each set of dots remained on the screen until the child responded. The stimuli were designed to avoid template matching and to prevent the total dot area being used as a cue to numerosity. Therefore the total area occupied by the dots was equivalent across displays (for a complete description of this experiment, see Guarini et al., 2014, 2020). We decided to range dots 1–9 according to other studies conducted with children attending kindergarten (Lyons et al., 2018). The measures considered were accuracy (i.e., number of correct answers) and mean reaction time for correct answers.

A regression analysis with simple RTs as the independent variable and dot RTs as the dependent variable was performed, and standardized residuals were used in the following analyses in order to obtain a measure of speed in magnitude comparison task that was free of the influence of general processing speed. Standard residuals and mean accuracy scores were used in the analyses.

#### Parents’ Assessment

##### Home numeracy

The children’s Home Numeracy was assessed through a questionnaire that was designed to be administered to the child’s parents (see Appendix 1). The questionnaire includes seven items that investigate the child’s home numeracy habits and skills (‘Do activities that require placing objects in order of size or length’) and their knowledge of numbers (‘Read or write numbers’). The reliability index was Cronbach’s α = 0.78. The responses were provided on a 4-point Likert scale, ranging from ‘Never’ to ‘Very often,’ mean scores were used in the analyses (minimum = 1, maximum = 5).

##### General cognitive ability

###### Non-verbal IQ

Parents, similarly to children, were administered the *Matrices* subtest of K-BIT 2 (Kaufman and Kaufman, 2004; Bonifacci and Nori, 2016). The test measures Non-Verbal IQ; it has different starting points based on the participant’s age and stops after four consecutive wrong responses. The maximum score is 46. Raw scores were converted into standard scores according to the Italian test manual. Split-half reliability coefficient in adult age (19–90 years) was 0.87.

##### Calculation skills

Parents were administered three subtests of the BDE-2 test (Biancardi et al., 2016): Quick Calculation, Approximate Calculation, and Written Calculation. The test’s manual offers a reliability index for the three main factors investigated, identified by factorial analysis. In addition, the Mental Calculation subtest from the MT battery was administered to parents (Cornoldi et al., 2010). Raw scores were used in the correlation analyses. Z-scores derived from the sample of the study were used for the Composite Numeracy Score.

###### Quick calculation

In this test, parents have 2 min to write the correct results of as many mixed operations as possible (additions, subtractions, multiplications, divisions) up to a maximum of 40. The score is the total number of correct answers they give in 2 min. The level of saturation of these items on the First Factor of the test was 0.80 and the scale’s Cronbach’s alpha of the First Factor was 0.74.

###### Approximate calculation

Parents have 2 min to indicate the correct result of 18 operations, indicating one out of four options. For example, the operation is 75:5 and they have to choose between 80, 375, 15, or 5. The score is the total of correct answers and the maximum score is 18. The level of saturation of these items on the First Factor of the test was 0.77 and the scale’s Cronbach’s alpha of the First Factor was 0.74.

###### Written calculation

Parents have 2 min to indicate the correct result of six written operations: two additions, two subtractions, and two multiplications (example: 356 + 579; 102−48; 216 × 29). The score is the total of correct answers and the maximum score is 6. The level of saturation of this item on the Second Factor of the test was 0.29 and the scale’s Cronbach’s alpha of the Second Factor was 0.62.

###### Mental calculation

The examiner reads 8 operations (2 additions, 2 subtractions, 2 multiplications, and 2 divisions) and parents have 60 s to answer each operation with the correct result. The score is the total of correct answers they give. The maximum score is 8. The test–retest reliability was 0.75.

A Composite Numeracy Score was computed by calculating mean z scores, derived from the study sample, for the Quick Calculation, Approximate Calculation, Written Calculation, and Mental Calculation.

##### ANS

###### Estimation

This task was developed on E-Prime for the purpose of the present study and adapted from Knops et al. (2014). Different sets of black dots were presented on a white circle against a black background. The numerosities were 10, 16, 24, 32, 48, 56, or 64 dots. Each numerosity was presented five times, each time in a different configuration, so that the same numerosity never appeared in consecutive trials. Participants were instructed to look at the circle with black dots inside for 500 ms and then to estimate the quantity of dots shown on the computer screen, typing the number using the keyboard. The mean distance between the correct number and the given number (differential) was calculated. Z-scores were computed on study sample.

###### Non-symbolic magnitude comparison task

In this task, parents were instructed to compare two sets of violet squares, which were simultaneously presented in two black rectangles on the left and right side of the screen, and they were instructed to choose the side with the larger numerosity by pressing a key that was congruent (left or right). The task was adapted from Landerl et al. (2009). Forty pairs of sets of squares were presented. The difficulty in making this decision is manipulated by varying the ratio or the numerical distance between the two sets. Each display consisted of between 20 and 72 squares, with the difference between the two displays ranging from 10 to 29 squares. To avoid the displays with the larger numerosity systematically consisting of smaller squares, each display included squares of different sizes. The maximum score is 40. The percentage of correct responses was used in the analyses and then z-scores were used to compute the composite score.

A composite ANS score was computed by calculating mean z scores, derived from the study sample, for the estimation and the non-symbolic magnitude comparison tasks.

### Data Analysis

Pearson correlations were performed to (aim 1) analyze associations between children’s early math skills and: environmental variables (SES and home numeracy), parents’ variables (non-verbal IQ, calculation skills, ANS), inter (non-verbal IQ, cognitive skills, linguistic skills) and intra-domain (ANS) children’s skills.

Then, in order to (aim 2) investigate predictors of children’s early math skills, as represented by the Composite Numeracy Score, a 3-step hierarchical regression analysis was run. Environmental and parents’ variables were included in the first step: home numeracy, parents’ calculation skills and ANS. In the second step, composite scores of children’s cognitive and language skills were included. Finally, in the last step, scores related to children’s ANS were included.

## Results

Descriptives for children’s and parents’ variables are showed in Table 1.

**TABLE 1:** Descriptive statistics for all the variables regarding children and parents.

**Group**	**Measures**	**Mean**	**SD**	**Minimum**	**Maximum**
Environmental	Home Numeracy*	3.0	0.6	2.0	5.0
variables	SES*	45.4	12.6	17.0	61.0
Children	Non-verbal IQ*	16.9	5.1	3.0	29.0
	Memory*	20.2	4.4	4.0	26.0
	Attention*	21.0	4.0	11.0	26.0
	Composite Cognitive Score°	0.33	0.59	–2.34	1.33
	Vocabulary*	33.6	2.3	24.0	36.0
	Phonological awareness*	3.8	2.6	0.0	6.0
	Composite Language Score°	0.12	0.72	–1.47	1.06
	Counting*	19.0	2.7	3.0	20.0
	Biunivocal Correspondence*	17.3	4.2	2.0	20.0
	Recognition*	8.2	1.9	1.0	9.0
	Reading of digits*	8.2	2.0	0.0	9.0
	Composite Numeracy Score°	0.60	0.54	–1.32	1.15
	Speed of processing^§^	531.3	148.0	280.2	826.0
	ANS_ Magnitude Comparison RTs^§^	1584.9	799.0	648.1	3417.2
	ANS- Magnitude Comparison Acc^	0.95	0.10	0.5	1.0
Parents	Non-verbal IQ*	37.7	5.1	20.0	46.0
	Quick calculation*	33.3	6.8	15.0	40.0
	Approximate Calculation*	15.5	2.3	9.0	18.0
	Written Calculation*	4.8	1.1	1.0	6.0
	Mental Calculation*	5.0	1.7	1.0	8.0
	Composite Numeracy Score*	0.01	0.75	–2.16	1.23
	ANS- Estimation (difference)*	11.9	7.7	4.7	52.5
	ANS-Magnitude comparison RTs^§^	1.4	0.4	0.8	2.6
	ANS-Magnitude comparison Acc^	0.96	0.5	0.68	1.0
	Composite ANS Score°	0.1	0.4	–1.42	0.97

*Raw scores;°*z*-scores; ^§^ reaction times; ^accuracy.

(1) The correlations between children’s early numeracy skills and the environmental and parents’ variables are detailed in Table 2A. A strong association was found between home numeracy and the Composite Numeracy Score (*r* = 0.399) as well as with the single tasks: biunivocal correspondence, recognition and reading of digits (excluded counting). On the counterpart, SES was not significantly related to children’s early numeracy skills (all *r* < 0.2). Considering the relationships between children’s early math skills and intergenerational patterns, there were no significant correlations with parents’ calculation skills (quick calculation, approximate calculation, written calculation, mental calculation). Considering parents’ ANS skills, there was only a marginal negative association between biunivocal correspondence and parents’ accuracy in magnitude comparison, as well as with the composite measure of parents’ ANS.

**TABLE 2A:** Pearson correlations coefficients (*r*) between environmental stimuli, intergenerational variables, and children’s skills.

		**Children**				
		**Counting**	**Biunivocal correspondence**	**Recognition of digits**	**Reading of digits**	**Composite Numeracy Score**
	SES	0.038	–0.135	–0.002	0.016	–0.052
	Home numeracy	0.170	0.268*	0.398**	0.340**	0.399**
	Non-verbal IQ	0.098	–0.097	0.193	0.164	0.095
	Quick calculation	0.054	0.004	0.047	0.078	0.058
	Approximate calculation	0.213	0.039	0.181	0.163	0.194
Parents	Written calculation	0.138	–0.238	–0.046	–0.108	–0.105
	Mental calculation	0.140	–0.022	0.077	0.110	0.094
	ANS-Estimation	–0.018	0.049	0.121	0.126	0.088
	ANS-Magnitude comparison (accuracy)	0.079	−0.253*	0.056	–0.002	–0.073
	ANS-Magnitude comparison (RTs)	–0.143	–0.106	–0.174	–0.212	0.212
	Composite ANS	0.064	−0.245^*a*^	–0.010	–0.091	–0.120
	Composite Numeracy Score	0.180	–0.071	0.086	0.081	0.079

***p < 0.01; *p < 0.05; ^*a*^*p* = 0.051 SES, socio-economic status; IQ, intellectual quotient; ANS, approximate number system; RT, reaction times.

In Table 2B, correlations between children’s cognitive, linguistic, and early numeracy skills are reported. A significant relationship was found between children’s non-verbal IQ and early math skills (Composite Numeracy Score as well as single tasks: counting, recognition and reading of digits, see Table 2B). Also, visuo-spatial memory skills were related to the Composite Numeracy Score and, in particular, with digit recognition and digit reading. The attention task was not specifically related to children’s early math skills (all *r* < 0.2), but the Composite Cognitive Score was related to all the early numeracy subtests and to the Composite Numeracy Score except for biunivocal correspondence. Language skills (Composite Language Score), and in particular phonological awareness, were mostly linked to recognition and reading of digits, whereas vocabulary was not significantly associated with children’s early number skills. Finally, as revealed in Table 2B, many significant correlations could be observed among ANS skills (RTs and accuracy) and the Composite Numeracy Score. Magnitude comparison accuracy, in particular, was strongly related to digit recognition and reading and the composite score (all *r* > 0.5).

**TABLE 2B:** Pearson correlations coefficients (*r*) among children’s variables.

	**Counting**	**Biunivocal correspondence**	**Recognition of digits**	**Reading of digits**	**Composite Numeracy Score**
Non-verbal IQ	0.272*	–0.020	0.325**	0.328**	0.278*
Memory	0.138	0.033	0.572**	0.516**	0.380**
Attention	0.041	–0.090	0.108	–0.012	0.001
Composite Cognitive Score	0.265*	–0.034	0.553**	0.456**	0.371**
Vocabulary	0.132	–0.191	0.208	0.217	0.082
Phonological awareness	0.160	–0.050	0.285*	0.391**	0.230
Composite Language Score	0.204	–0.144	0.349**	0.442**	0.236
ANS- Magnitude comparison (RTs)	–0.179	0.118	−0.410**	−0.449**	−0.258*
ANS- Magnitude comparison (accuracy)	0.246	0.337**	0.536**	0.574**	0.563**

**p < 0.01; *p < 0.05; IQ, intellectual quotient; RT, reaction times; ANS, approximate number system.

(2) Table 3 shows the results of the hierarchical analysis investigating predictors of children’s early math skills, as represented by the Composite Numeracy Score. For the first step, only home numeracy resulted as a significant predictor of children’s early math skills, and the model explained 20.4% of variance. At the second step, children’s cognitive and linguistic skills (Composite Scores) were also included as potential predictors, adding a portion of explained variance (7.6%) that tended to significance (*p* = 0.055): home numeracy, as well as children’s cognitive skills, resulted as significant predictors. Children’s magnitude comparison performance (RTs and accuracy) was added as an additional potential predictor at step 3, and the model reached a total explained variance of 39.9%. Home numeracy resulted, again, as a significant predictor; also, children’s accuracy in the dot comparison task significantly predicted their early math skills.

**TABLE 3:** Hierarchical regression; dependent variables: children’s composite numeracy score.

		**Non-standardized coefficients**		**Standardized coefficient**
**Step**		**B**	**SE B**	**β**
1∘ (*R*^2^ = 0.204)	Home numeracy	0.234	0.064	0.429**
	Composite ANS (Parents)	–0.171	0.149	–0.137
	Composite Numeracy Score (Parents)	0.147	0.087	0.204
2∘ (ΔR^2^ = 0.076, *p* = 0.055)	Home numeracy	0.193	0.065	0.355**
	Composite ANS (Parents)	–0.170	0.146	–0.136
	Composite Numeracy Score (Parents)	0.102	0.087	0.142
	Composite Cognitive Score (Children)	0.252	0.111	0.275*
	Composite Language Score (Children)	0.026	0.091	0.035
3∘ (ΔR^2^ = 0.119, *p* = 0.006)	Home numeracy	0.136	0.063	0.250*
	Composite ANS (Parents)	–0.102	0.138	–0.082
	Composite Numeracy Score (Parents)	0.066	0.081	0.092
	Composite Cognitive Score (Children)	0.143	0.109	0.156
	Composite Language Score (Children)	–0.017	0.093	–0.023
	Children’s magnitude comparison (accuracy)	2.181	0.672	0.399**
	Children’s magnitude comparison (RT)	–0.014	0.065	–0.027

**p < 0.01; *p < 0.05. ANS, approximate number system; RT, reaction times.

## Discussion

In the present study, we aimed to evaluate (1) the variables associated with early math skills in preschool children, as well as (2) concurrent predictors of children’s early math skills, including environmental variables (SES, home numeracy), intergenerational paths (parents’ non-verbal IQ, calculation skills, and ANS) and children’s cognitive, linguistic, and ANS skills. The main results are that, (1) regarding the analysis of associations, children’s early math skills were related to one aspect of the environment, namely home numeracy activities, but not to SES. Children’s early math skills did correlate with children’s cognitive and language skills, but not with parents’ calculation and ANS abilities, confirming our expectations only partially; (2) as far as the analysis of predictors is concerned, children’s non-symbolic magnitude comparison accuracy, as a measure of ANS, was the strongest predictor of their early math skills, but home numeracy also predicted children’s early math skills over and above a number of parent and child variables (e.g., cognitive skills, language skills).

Regarding the first aim, we found multiple relationships between parents’ and children’s skills, and within children’s skills. In terms of the associations between children’s early numeracy and environmental variables (SES, home numeracy), it is of interest that there was no significant relationship between SES and children’s skills. This result seems to be in contrast with the reported literature about the gap in math achievement of low-income children compared to high SES peers (Reardon and Portilla, 2016). However, as discussed in the introduction, some previous studies failed to find the achievement gap in low-SES children, at least for non-verbal math tasks (Jordan et al., 1992; Jordan and Levine, 2009). Baird (2012) found significant differences in the roles of SES on math skills between countries and different school systems. To this regard, for Italy, the OCSE report accounts for a minor predictive role of SES (Quintano et al., 2009). Therefore, according to Elliott and Bachman (2018), the relationship between SES and math skills should not be considered as a direct relationship, but rather to be mediated by how SES impacts on moderating factors such as beliefs, home numeracy, and math talk. In contrast, as we expected, a significant strong relationship between home numeracy activities and early math skills in children was described, reinforcing the strong evidence reported in literature about the important role of home numeracy in fostering numerical development in children (LeFevre et al., 2009). Therefore, results from the present study seem to suggest that home numeracy is more strongly associated with math skills than SES.

Concerning the role of the intergenerational path of math skills, neither parents’ ANS skills nor calculation were related to children’s formal math skills. This result was in contrast with our expectations and with previous studies (Braham and Libertus, 2017; Navarro et al., 2018).

Concerning the correlations between the children’s skills, a strong association between cognitive skills and early numeracy has been pointed out, revealing inter-domain relationships. Indeed, non-verbal IQ and visuo-spatial memory show a strict association with recognition and reading of digits, reinforcing the idea that math skills might be, at least in part, related to general intellectual functioning (Poletti, 2017), as well as to visuo-spatial memory skills (Cirino, 2011; Simmons et al., 2012; Zhang and Lin, 2015). It also has to be noticed that the association between non-verbal IQ and early numeracy might reflect an association between IQ and visual-spatial skills (as both non-verbal IQ and memory tests were highly confounded by visual-spatial skills; Cornoldi et al., 1995). In addition, the present study also gives useful insight into the relationship between linguistic and numeracy skills. As expected, there was a significant relationship between mean language scores and recognition and reading of digits (Purpura and Ganley, 2014; Korpipää et al., 2019), with phonological awareness playing a relevant role. Contrasting results were found regarding the relationship between phonological awareness and mathematical skills, and some authors have suggested that it might not be constant across development (Passolunghi et al., 2015).

Furthermore, a strict intra-domain relationship was observed, since non-symbolic magnitude comparison tasks were significantly related to early math skills, in line with previous evidence regarding the foundational role of ANS skills in number development (Libertus et al., 2013).

Taken as a whole, the results from the correlational analyses provided interesting insight into the complex pattern of relationships within and between groups, highlighting the fact that, beyond a relationship between early math skills and ANS-related measures, early numeracy skills develop within a network of multiple relationships, involving both environmental and within-subject variables.

In order to better understand the strength of these multiple factors, we developed as second aim a regression model that included, as the first step, the environmental and intergenerational variables, as the second step, the children’s cognitive and linguistic profile, and as the third step the children’s ANS skills. It emerged that environmental variables alone explained around 20% of the variance and home numeracy resulted as a significant predictor of children’s early math skills. By contrast, no effect of intergenerational variables (parents’ calculation and ANS skills) was found. The second step of the analysis added a marginally significant proportion of variance showing that children’s cognitive, but not linguistic skills predicted early numeracy skills. Although in the correlation analyses it was found a significant relationship between phonological awareness and early math skills and previous literature strongly reports evidence of language as a predictor of numeracy skills, in the regression analysis the children’s linguistic profile did not emerge as a significant concurrent predictor of their early numeracy skills, namely it did not add a significant proportion of explained variance in the model. A possible explanation is that, within a complex model that accounts for many different variables and in a sample of this age, the role of language skills becomes secondary to other skills and, particularly, to home numeracy activities. Further investigation through multiple-variables models would be necessary to better disentangle the role of language in early math skills when other environmental and cognitive variables are taken into account. Finally, we wanted to evaluate whether children’s ANS-related skills represented a meaningful predictor of their numeracy skills above the role of environmental and cognitive factors. The variance added in the third step was significant and accuracy scores in the dot comparison task, together with home numeracy, were the significant predictors of early numeracy in the final model.

Results from the regression analysis, therefore, highlighted the main role played by home numeracy and children’s ANS skills, whereas parents’ math skills and children’s cognitive and linguistic measures, although partially related to early math skills, resulted in being secondary factors. This picture is in line with previous studies that highlighted, on one side, the role of home numeracy (e.g., Kleemans et al., 2012) and, on the other hand, that of children’s non-symbolic magnitude comparison skills (Libertus et al., 2011). However, few previous studies have considered these factors together and the present study offers original insight into the possibility that both these factors are similarly related to children’s early math skills.

On the contrary, the pattern of results that emerges from the present study is partially in contrast with previous evidence of a direct relationship between parents’ and children’s math skills (Braham and Libertus, 2017; Navarro et al., 2018). It is possible that the intergeneration pattern of math skills is weaker than that of literacy skills, as reported in many previous studies (e.g., van Bergen et al., 2014). In other words, although some evidence suggests that math skills might have genetic/biological markers (Docherty et al., 2010), and that math disorders might be a “familiar” disorder (Shalev et al., 2001), it may be that in the pathway to the behavioral level many more external variables play a role. It has also to be considered that genetic links might be dependent on specific samples and situations: previous study on heritability estimates, which, however, found contrasting results, included children from clinical samples (Ludwig et al., 2013; Pettigrew et al., 2015). The present study was, instead, conducted on a sample of typically developing children and the genetic influence of math skills might result weaker. Further, it is known that many children (and adults) may underperform in math tasks because of stereotype threats (Tomasetto et al., 2011), math anxiety (Maloney et al., 2015), or didactic opportunities (Slavin et al., 2009; Pellizzoni et al., 2020). Considering the paucity of research on this issue, further investigation is warranted to better understand the intergenerational pathway of math skills and of possible intervening variables.

Finally, children’s cognitive skills were related to their early numeracy skills, but when their ANS skills were considered, the latter became the unique significant individual predictor of children’s early math skills. In our view, this pattern is not in contrast with previous studies that documented relationships between cognitive, linguistic, and math skills, but should be interpreted as a suggestion that these cross-domain connections should not be considered as strong as domain-specific variables.

These results represent an original picture of the complex interplay amongst variables involved in the development of early math skills. We previously reviewed evidence regarding the consolidated dual relationship between home numeracy and early math skills, and between ANS measures and early numeracy development. However, the present study offers a new window on this literature, taking account of these different variables altogether and adding the assessment of parents’ math skills. Undoubtedly, this line of research needs a more in-depth investigation in light of the limitations of the present study that might limit the generalizability of results. First, the sample is relatively small and data were collected in a concurrent design, therefore further investigation on a wider sample and through longitudinal design is needed. This would also allow alternative possibilities for the directions of relationships found in the present study, such as, for example, the role that children’s early math skills could have in eliciting more offers of home numeracy activities by their parents (i.e., evocative biology-environment correlation; Plomin et al., 1977), or the role of home numeracy activities as a mediator between parents’ and children’s math skills. Secondly, there is debate in the literature about which ANS tasks have the highest validity (Smets et al., 2014) and replication is required with different types of tasks, both in relation to ANS-related skills and early cognitive, literacy, and math skills. In particular, the magnitude comparison task proposed to preschool children ranged from 1–9, included the subitizing range (1–4) with possible contamination of the ANS measure (Lyons et al., 2018). Thus, it would be recommended to replicate our results by avoiding the subitizing range. In addition, as far as older children are concerned, symbolic magnitude comparison tasks could be included, in order to better understand the differential role of symbolic and non-symbolic measures in math skills. With reference to the relationship between SES and math skills, it is possible that the variance in the present sample was not sufficiently wide to detect differential effects. Furthermore, some limitations on the measured used should be taken into account: despite the home numeracy questionnaire showed good reliability, no factor analysis was performed because of the small sample; also, reliability measures are missing for some of the experimental tasks and for composite scores. Finally, the present study did not consider factors such as parents’ attitudes and expectations related to their children’s math performance (e.g., Neuenschwander et al., 2007), as well as familiar variables such as children’s birth order that could impact on the time their parents spend in doing activities with them (e.g., Price, 2008); these variables should be included in future research. Within this framework, it has also to be noticed that in the present study, as in others that adopted a similar methodology (see Braham and Libertus, 2017; Navarro et al., 2018), the term intergenerational is used, but selection procedures did not include all parents (father and mothers) and there is no direct control of genetic impact. Therefore, the term intergenerational, although frequently adopted in the literature on the relationships between parents’ and children’s cognitive (math, literacy) skills, should be interpreted with caution and more studies involving both parents are needed.

Despite these limitations, the suggestion that came from the present study is that the type of activity that parents carry out in the home environment might be more powerful than their actual efficiency in math skills. In terms of children’s skills, moreover, the present study showed that domain-specific skills, such as those related to the ANS, are more important than domain-general cognitive and linguistic skills in shaping early numeracy competence. Therefore, stimulating children’s ANS skills is of importance for favoring their early math skills. Further research in other age ranges (primary and secondary school) should better investigate the developmental patterns of complex interactions across individual and environmental variables in predicting math skills. For example, in a similar research design, Bernabini et al. (2020) found that, in fifth graders, children’s symbolic comparison skills were the most significant predictor of their math skills, above and beyond mothers’ math skills.

Finally, the present study suggests important implications for the educational setting, where it is important to activate both direct (directed at the child) and indirect (directed at parents) instruction in numeracy development. Regarding the direct instruction approach, training programs aimed at improving ANS skills in young children (e.g., Van Herwegen et al., 2017) are recommended. Considering interventions directed at parents, a few past studies have documented the efficacy of training to improve their ability to involve their children in adequate home numeracy activities (e.g., Niklas et al., 2016); our study suggests that this type of intervention should be implemented in order to improve home numeracy in families of preschoolers.

## Data Availability Statement

The datasets generated for this study are available on request to the corresponding author.

## Ethics Statement

The studies involving human participants were reviewed and approved by Bioethical Committee University of Bologna. Written informed consent to participate in this study was provided by the participants’ legal guardian/next of kin.

## Author Contributions

LB and PB conceived the presented idea and developed the assessment protocol. LB carried out the experiment, collected and analyzed data, and wrote a preliminary version of the manuscript. LB, PB, and VT performed statistical analysis. AG contributed to the interpretation of the results. AG, VT, and PB revised and significantly contributed to the final version of the manuscript. All authors discussed the results and contributed to the final manuscript.

## Conflict of Interest

The authors declare that the research was conducted in the absence of any commercial or financial relationships that could be construed as a potential conflict of interest.

## Appendix

**Appendix 1 |** Questionnaire for parents on home numeracy activities.

### Instructions

**TABLE 5:** delete it

We ask you to think about your child’s behavior. How many times does your child do the following activities at home? Rate your responses on the scale detailed below, from 1 (never) to 5 (everyday).^∗^						

		**Never**	**Sometimes**	**Often**	**Very Often**	**Every day**
1	Count objects	1	2	3	4	5
2	Read or write numbers	1	2	3	4	5
3	Use games (even on Tablet or PC) that use numbers	1	2	3	4	5
4	Use games that use dice	1	2	3	4	5
5	Repeat songs that contain numbers	1	2	3	4	5
6	Do activities that require the ordering of size or length of objects	1	2	3	4	5
7	Do simple calculations (2 + 1 = 3) in games or during other daily activities	1	2	3	4	5
						

## References

[B1] Adams T. L. (2003). Reading mathematics: more than words can say. *Read. Teach.* 56 786–795.

[B2] American Psychiatric Association, and Taskforce on Dsm-5 (2013). *Diagnostic and Statistical Manual of Mental Disorders*, 5th Edn Washington, DC: American Psychiatric Association.

[B3] Baird K. (2012). Class in the classroom: the relationship between school resources and math performance among low socio-economic status students in 19 rich countries. *Educ. Econ.* 20 484–509. 10.1080/09645292.2010.511848

[B4] Baron-Cohen S. Murphy L. Chakrabarti B. Craig I. Mallya U. Lakatošová S. (2014). A genome wide association study of mathematical ability reveals an association at chromosome 3q29, a locus associated with autism and learning difficulties: a preliminary study. *PLoS One* 9:e0096374. 10.1371/journal.pone.0096374 24801482 PMC4011843

[B5] Bernabini L. Tobia V. Bonifacci P. (2020). Intergenerational features of math skills: symbolic and nonsymbolic magnitude comparison and written calculation in mothers and children. *J. Cogn. Dev.* (in press).

[B6] Biancardi A. Nicoletti C. Bachmann C. (2016). *BDE 2-Batteria Discalculia Evolutiva: Test per la Diagnosi dei Disturbi Dell’elaborazione Numerica e del Calcolo in età Evolutiva–8-13 Anni.* Trento: Edizioni Centro Studi Erickson.

[B7] Blevins-Knabe B. Austin A. B. Musun L. Eddy A. Jones R. M. (2000). Family home care providers’ and parents’ beliefs and practices concerning mathematics with young children. *Early Child Dev. Care* 165 41–58. 10.1080/0300443001650104

[B8] Bonifacci P. Montuschi M. Lami L. Snowling M. J. (2014). Parents of children with dyslexia: cognitive, emotional and behavioural profile. *Dyslexia* 20 175–190. 10.1002/dys.1469 24265066

[B9] Bonifacci P. Nori R. (2016). *KBIT-2. Kaufman Brief Intelligence Test Second Edition. Contributo alla taratura italiana [contribution to italian standardization].* Firenze: Giunti-OS.

[B10] Bonifacci P. Pellizzari C. Giuliano P. Serra P. (2015). *IDA - Indicatori delle Difficoltà di Apprendimento.* Florence: Hogrefe editore.

[B11] Bonifacci P. Snowling M. J. (2008). Speed of processing and reading disability: a cross-linguistic investigation of dyslexia and borderline intellectual functioning. *Cognition* 107 999–1017. 10.1016/j.cognition.2007.12.006 18272144

[B12] Bonifacci P. Tobia V. Bernabini L. Marzocchi G. M. (2016). Early literacy and numeracy skills in bilingual minority children: toward a relative independence of linguistic and numerical processing. *Front. Psychol.* 7:1020. 10.3389/fpsyg.2016.01020 27458413 PMC4935724

[B13] Bradley R. H. Corwyn R. F. (2002). Socio-economic status and child development. *Annu. Rev. Psychol.* 53 371–399. 10.1146/annurev.psych.53.100901.135233 11752490

[B14] Braham E. J. Libertus M. E. (2017). Intergenerational associations in numerical approximation and mathematical abilities. *Dev. Sci.* 20:e12436. 10.1111/desc.12436 27496658 PMC6035787

[B15] Braham E. J. Libertus M. E. McCrink K. (2018). Children’s spontaneous focus on number before and after guided parent–child interactions in a children’s museum. *Dev. Psychol.* 54 1492–1492. 10.1037/dev0000534 30047774 PMC6132254

[B16] Brannon E. M. Merritt D. J. (2011). “evolutionary foundations of the approximate number system,” in *Space, Time and Number in the Brain*, eds Dehaene S. Brannon E. (Amsterdam: Elsevier), 207–224. 10.1016/B978-0-12-385948-8.00014-1

[B17] Carroll J. M. Holliman A. J. Weir F. Baroody A. E. (2019). Literacy interest, home literacy environment and emergent literacy skills in preschoolers. *J. Res. Read.* 42 150–161. 10.1111/1467-9817.12255

[B18] Caspi A. Moffitt T. E. (2006). Gene-environment interactions in psychiatry: joining forces with neuroscience. *Nat. Rev. Neurosci.* 7 583–590. 10.1038/nrn1925 16791147

[B19] Cirino P. T. (2011). The interrelationships of mathematical precursors in kindergarten. *J. Exp. Child Psychol.* 108 713–733. 10.1016/j.jecp.2010.11.004 21194711 PMC3043138

[B20] Cornoldi C. Pra Baldi A. Friso G. Giacomin A. Giofrà D. Zaccaria S. (2010). *Prove di lettura MT Avanzate - 2. Prove MT Avanzate di Lettura e Matematica 2 per il biennio della Scuola Secondaria di II grado.* Firenze: Giunti-OS.

[B21] Cornoldi C. Vecchia R. D. Tressoldi P. E. (1995). Visuo-spatial working memory limitations in low visuo-spatial high verbal intelligence children. *J. Child Psychol. Psychiatry* 36 1053–1064. 10.1111/j.1469-7610.1995.tb01350.x 7593398

[B22] Cragg L. Keeble S. Richardson S. Roome H. E. Gilmore C. (2017). Direct and indirect influences of executive functions on mathematics achievement. *Cognition* 162 12–26. 10.1016/j.cognition.2017.01.014 28189034

[B23] Davis O. S. Band G. Pirinen M. Haworth C. M. Meaburn E. L. Kovas Y. (2014). The correlation between reading and mathematics ability at age twelve has a substantial genetic component. *Nat. Commun.* 5 1–6. 10.1038/ncomms5204 25003214 PMC4102107

[B24] De Smedt B. Noël M. P. Gilmore C. Ansari D. (2013). How do symbolic and nonsymbolic numerical magnitude processing skills relate to individual differences in children’s mathematical skills? A review of evidence from brain and behavior. *Trends Neurosci. Educ.* 2 48–55. 10.1016/j.tine.2013.06.001

[B25] Dehaene S. (1992). Varieties of numerical abilities. *Cognition* 44 1–42. 10.1016/0010-0277(92)90049-N 1511583

[B26] Dehaene S. (1997). “Babies who count,” in *The Number Sense: How the Mind Creates Mathematics.* New York: Oxford University Press (OUP), 41–63.

[B27] Desoete A. Praet M. Titeca D. Ceulemans A. (2013). Cognitive phenotype of mathematical learning disabilities: what can we learn from siblings? *Res. Dev. Disabil.* 34 404–412. 10.1016/j.ridd.2012.08.022 23023299

[B28] Docherty S. J. Kovas Y. Petrill S. A. Plomin R. (2010). Generalist genes analysis of DNA markers associated with mathematical ability and disability reveals shared influence across ages and abilities. *BMC Genet.* 11:61. 10.1186/1471-2156-11-61 20602751 PMC2909150

[B29] Duncan G. J. Magnuson K. (2011). “The nature and impact of early achievement skills, attention skills, and behavior problems,” in *Whither Opportunity*, eds Duncan G. J. Murnane R. J. (Lawrence, NY: Russell Sage), 47–70.

[B30] Elliott L. Bachman H. J. (2018). SES disparities in early math abilities: the contributions of parents’ math cognitions, practices to support math, and math talk. *Dev. Rev.* 49 1–15. 10.1016/j.dr.2018.08.001

[B31] Elliott L. Braham E. J. Libertus M. E. (2017). Understanding sources of individual variability in parents’ number talk with young children. *J. Exp. Child Psychol.* 159 1–15. 10.1016/j.jecp.2017.01.011 28266331

[B32] Espy K. A. McDiarmid M. M. Cwik M. F. Stalets M. M. Hamby A. Senn T. E. (2004). The contribution of executive functions to emergent mathematic skills in preschool children. *Dev. Neuropsychol.* 26 465–486. 10.4324/9780203764428-6 15276905

[B33] Feigenson L. Dehaene S. Spelke E. (2004). Core systems of number. *Trends Cogn. Sci.* 8 307–314. 10.1016/j.tics.2004.05.002 15242690

[B34] Fuhs M. W. McNeil N. M. (2013). ANS acuity and mathematics ability in preschoolers from low-income homes: contributions of inhibitory control. *Dev. Sci.* 16 136–148. 10.1111/desc.12013 23278935

[B35] Gilmore C. Attridge N. Clayton S. Cragg L. Johnson S. Marlow N. (2013). Individual differences in inhibitory control, not non-verbal number acuity, correlate with mathematics achievement. *PLoS One* 8:e67374. 10.1371/journal.pone.0067374 23785521 PMC3681957

[B36] Göbel S. M. Snowling M. J. (2010). Number-processing skills in adults with dyslexia. *Q. J. Exp. Psychol.* 63 1361–1373. 10.1080/17470210903359206 19899014

[B37] Göbel S. M. Watson S. E. Lervåg A. Hulme C. (2014). Children’s arithmetic development: it is number knowledge, not the approximate number sense, that counts. *Psychol. Sci.* 25 789–798. 10.1177/0956797613516471 24482406

[B38] Goffin C. Ansari D. (2019). How are symbols and nonsymbolic numerical magnitudes related? Exploring bidirectional relationships in early numeracy. *Mind Brain Educ.* 13 143–156. 10.1111/mbe.12206

[B39] Gordon P. (2004). Numerical cognition without words: evidence from Amazonia. *Science* 306 496–499. 10.1126/science.1094492 15319490

[B40] Guarini A. Sansavini A. Fabbri M. Alessandroni R. Faldella G. Karmiloff-Smith A. (2014). Basic numerical processes in very preterm children: a critical transition from preschool to school age. *Early Hum. Dev.* 90 103–111. 10.1016/j.earlhumdev.2013.11.003 24331582

[B41] Guarini A. Tobia V. Bonifacci P. Faldella G. Sansavini A. (2020). Magnitude comparisons, number knowledge and calculation in verypreterm children and children with specific learning disability: a cross-population study using eye-tracking. *J. Learn. Disabil.* 22219420950651 (in press). 10.1177/0022219420950651 32814504

[B42] Halberda J. Feigenson L. (2008). Developmental change in the acuity of the “Number Sense”: the approximate number system in 3-, 4-, 5-, and 6-year-olds and adults. *Dev. Psychol.* 44 1457–1465. 10.1037/a0012682 18793076

[B43] Haworth C. M. A. Wright M. J. Martin N. W. Martin N. G. Boomsma D. I. Bartels M. (2009). A twin study of the genetics of high cognitive ability selected from 11,000 twin pairs in six studies from four countries. *Behav. Genet.* 39 359–370. 10.1007/s10519-009-9262-3 19381794 PMC2740717

[B44] Hill H. C. Rowan B. Ball D. L. (2005). Effects of teachers’ mathematical knowledge for teaching on student achievement. *Am. Educ. Res. J.* 42 371–406. 10.3102/00028312042002371

[B45] Hollingshead A. B. (2011). Four factor index of social status. *Yale J. Sociol.* 8 21–51.

[B46] Izard V. Sann C. Spelke E. S. Streri A. (2009). Newborn infants perceive abstract numbers. *Proc. Natl. Acad. Sci. U.S.A.* 106 10382–10385. 10.1073/pnas.0812142106 19520833 PMC2700913

[B47] Jordan N. C. Huttenlocher J. Levine S. C. (1992). Differential calculation abilities in young children from middle-and low-income families. *Dev. Psychol.* 28 644–653.

[B48] Jordan N. C. Kaplan D. Ramineni C. Locuniak M. N. (2009). Early math matters: kindergarten number competence and later mathematics outcomes. *Dev. Psychol.* 45 850–867. 10.1037/a0014939 19413436 PMC2782699

[B49] Jordan N. C. Levine S. C. (2009). Socio-economic variation, number competence, and mathematics learning difficulties in young children. *Dev. Disabil. Res. Rev.* 15 60–68. 10.1002/ddrr.46 19213011

[B50] Kaufman A. S. Kaufman N. L. (2004). *Kaufman Brief Intelligence Test*, 2nd Edn NCS Pearson, Inc.

[B51] Kleemans T. Peeters M. Segers E. Verhoeven L. (2012). Child and home predictors of early numeracy skills in kindergarten. *Early Childh. Res. Q.* 27 471–477. 10.1016/j.ecresq.2011.12.004

[B52] Kleemans T. Segers E. Verhoeven L. (2016). “Towards a theoretical framework on individual differences in numerical abilities: role of home numeracy experiences,” in *Early Childhood Mathematics Skill Development in the Home Environment*, eds Blevins-Knabe B. Austin A. M. B. (Cham: Springer), 71–86.

[B53] Knops A. Dehaene S. Berteletti I. Zorzi M. (2014). Can approximate mental calculation account for operational momentum in addition and subtraction? *Q. J. Exp. Psychol.* 67 1541–1556. 10.1080/17470218.2014.890234 24499435

[B54] Korkman M. Kirk U. Kemp S. (2014). *NEPSY-II.* London: Pearson.

[B55] Korpipää H. Niemi P. Aunola K. Koponen T. Hannula-Sormunen M. Stolt S. (2019). Prematurity and overlap between reading and arithmetic: the cognitive mechanisms behind the association. *Contemp. Educ. Psychol.* 56 171–179. 10.1016/j.cedpsych.2019.01.005

[B56] Landerl K. Fussenegger B. Moll K. Willburger E. (2009). Dyslexia and dyscalculia: two learning disorders with different cognitive profiles. *J. Exp. Child Psychol.* 103 309–324. 10.1016/j.jecp.2009.03.006 19398112

[B57] LeFevre J. A. Fast L. Skwarchuk S. L. Smith-Chant B. L. Bisanz J. Kamawar D. (2010). Pathways to mathematics: longitudinal predictors of performance. *Child Dev.* 81 1753–1767. 10.1111/j.1467-8624.2010.01508.x 21077862

[B58] LeFevre J. A. Kwarchuk S. L. Smith-Chant B. L. Fast L. Kamawar D. Bisanz J. (2009). Home numeracy experiences and children’s math performance in the early school years. *Can. J. Behav. Sci.* 41 55–66. 10.1037/a0014532

[B59] Leibovich T. Ansari D. (2016). The symbol-grounding problem in numerical cognition: a review of theory, evidence, and outstanding questions. *Can. J. Exp. Psychol. Rev. Can. Psychol. Exp.* 70 12–23. 10.1037/cep0000070 26913782

[B60] Levine S. C. Suriyakham L. W. Rowe M. L. Huttenlocher J. Gunderson E. A. (2010). What counts in the development of young children’s number knowledge? *Dev. Psychol.* 46 1309–1319. 10.1037/a0019671 20822240 PMC2998540

[B61] Libertus M. E. Feigenson L. Halberda J. (2011). Preschool acuity of the approximate number system correlates with school math ability. *Dev. Sci.* 14 1292–1300. 10.1111/j.1467-7687.2011.01080.x 22010889 PMC3338171

[B62] Libertus M. E. Feigenson L. Halberda J. (2013). Is approximate number precision a stable predictor of math ability? *Learn. Individ. Differ.* 25 126–133. 10.1016/j.lindif.2013.02.001 23814453 PMC3692560

[B63] Ludwig K. U. Sämann P. Alexander M. Becker J. Bruder J. Moll K. (2013). A common variant in Myosin-18B contributes to mathematical abilities in children with dyslexia and intraparietal sulcus variability in adults. *Transl. Psychiatry* 3:e229. 10.1038/tp.2012.148 23423138 PMC3591001

[B64] Lyons I. M. Bugden S. Zheng S. De Jesus S. Ansari D. (2018). Symbolic number skills predict growth in nonsymbolic number skills in kindergarteners. *Dev. Psychol.* 54 440–457. 10.1037/dev0000445 29154653

[B65] Maloney E. A. Ramirez G. Gunderson E. A. Levine S. C. Beilock S. L. (2015). Intergenerational effects of parents’ math anxiety on children’s math achievement and anxiety. *Psychol. Sci.* 26 1480–1488. 10.1177/0956797615592630 26253552

[B66] Manolitsis G. Georgiou G. K. Tziraki N. (2013). Examining the effects of home literacy and numeracy environment on early reading and math acquisition. *Early Childh. Res. Q.* 28 692–703. 10.1016/j.ecresq.2013.05.004

[B67] Mazzocco M. M. M. Feigenson L. Halberda J. (2011). Preschoolers’ precision of the approximate number system predicts later school mathematics performance. *PLoS One* 6:e0023749. 10.1371/journal.pone.0023749 21935362 PMC3173357

[B68] Mutaf Yildiz B. Sasanguie D. De Smedt B. Reynvoet B. (2018a). Frequency of home numeracy activities is differentially related to basic number processing and calculation skills in kindergartners. *Front. Psychol.* 9:340. 10.3389/fpsyg.2018.00340 29623055 PMC5874519

[B69] Mutaf Yildiz B. Sasanguie D. De Smedt B. Reynvoet B. (2018b). Investigating the relationship between two home numeracy measures: a questionnaire and observations during Lego building and book reading. *Br. J. Dev. Psychol.* 36 354–370. 10.1111/bjdp.12235 29393519

[B70] Navarro M. G. Braham E. J. Libertus M. E. (2018). Intergenerational associations of the approximate number system in toddlers and their parents. *Br. J. Dev. Psychol.* 36 521–539. 10.1111/bjdp.12234 29377230

[B71] Neuenschwander M. P. Vida M. Garrett J. L. Eccles J. S. (2007). Parents’ expectations and students’ achievement in two western nations. *Int. J. Behav. Dev.* 31 594–602. 10.1177/0165025407080589

[B72] Nieder A. Dehaene S. (2009). Representation of number in the brain. *Annua. Rev. Neurosci.* 32 185–208. 10.1146/annurev.neuro.051508.135550 19400715

[B73] Niklas F. Cohrssen C. Tayler C. (2016). Improving preschoolers’ numerical abilities by enhancing the home numeracy environment. *Early Educ. Dev.* 27 372–383. 10.1080/10409289.2015.1076676

[B74] Niklas F. Schneider W. (2014). Casting the die before the die is cast: the importance of the home numeracy environment for preschool children. *Eur. J. Psychol. Educ.* 29 327–345.

[B75] Oliver B. Harlaar N. Thomas M. E. H. Kovas Y. Walker S. O. Petrill S. A. (2004). A twin study of teacher-reported mathematics performance and low performance in 7- year-olds. *J. Educ. Psychol.* 96 504–517.

[B76] Passolunghi M. C. Lanfranchi S. Altoè G. Sollazzo N. (2015). Early numerical abilities and cognitive skills in kindergarten children. *J. Exp. Child Psychol.* 135 25–42. 10.1016/j.jecp.2015.02.001 25818537

[B77] Pellizzoni S. Apuzzo G. M. De Vita C. Agostini T. Ambrosini M. Passolunghi M. C. (2020). Exploring EFs and math abilities in highly deprived contexts. *Front. Psychol.* 11:383. 10.3389/fpsyg.2020.00383 32210893 PMC7076912

[B78] Pennington B. (2006). From single to multiple deficit models of developmental disorders. *Cognition* 101 385–413. 10.1016/j.cognition.2006.04.008 16844106

[B79] Pettigrew K. A. Fajutrao Valles S. F. Moll K. Northstone K. Ring S. Pennell C. (2015). Lack of replication for the myosin-18B association with mathematical ability in independent cohorts. *Genes Brain Behav.* 14 369–376. 10.1111/gbb.12213 25778778 PMC4672701

[B80] Plomin R. DeFries J. C. Loehlin J. C. (1977). Genotype-environment interaction and correlation in the analysis of human behavior. *Psychol. Bull.* 84 309–322. 10.1037/0033-2909.84.2.309 557211

[B81] Poletti M. (2017). Profilo WISC-IV nella discalculia evolutiva e relazione tra WISC-IV e BDE in un campione clinico di bambini con difficoltà di apprendimento o con disturbi specifici di apprendimento. *Psicol. Clin. Dello Svil.* 21 105–120. 10.1449/86187

[B82] Price J. (2008). Parent-child quality time does birth order matter? *J. Hum. Resour.* 43 240–265.

[B83] Purpura D. Schmitt S. Ganley C. (2017). Foundations of mathematics and literacy: the role of executive functioning components. *J. Exp.Child Psychol.* 153 15–34. 10.1016/j.jecp.2016.08.010 27676183

[B84] Purpura D. J. Ganley C. M. (2014). Working memory and language: skill-specific or domain-general relations to mathematics? *J. Exp. Child Psychol.* 122 104–121. 10.1016/j.jecp.2013.12.009 24549230

[B85] Purpura D. J. Hume L. E. Sims D. M. Lonigan C. J. (2011). Early literacy and early numeracy: the value of including early literacy skills in the prediction of numeracy development. *J. Exp. Child Psychol.* 110 647–658. 10.1016/j.jecp.2011.07.004 21831396

[B86] Quintano C. Castellano R. Longobardi S. (2009). L’influenza dei fattori socio economici sulle competenze degli studenti italiani. Un’analisi multilevel dei dati PISA 2006. *Riv. Econ. Stat. Territ*. 2, 109–149. 10.3280/REST2009-002004

[B87] Raghubar K. P. Barnes M. A. Hecht S. A. (2010). Working memory and mathematics: a review of developmental, individual difference, and cognitive approaches. *Learn. Individ. Differ.* 20 110–122. 10.1016/j.lindif.2009.10.005

[B88] Reardon S. F. Portilla X. A. (2016). Recent trends in income, racial, and ethnic school readiness gaps at kindergarten entry. *Aera Open* 2:233285841665734 10.1177/2332858416657343

[B89] Schmitt S. A. Geldhof G. J. Purpura D. J. Duncan R. McClelland M. M. (2017). Examining the relations between executive function, math, and literacy during the transition to kindergarten: a multi-analytic approach. *J. Educ. Psychol.* 109 1120–1140. 10.1037/edu0000193

[B90] Sénéchal M. LeFevre J. A. (2001). Storybook reading and parent teaching: links to language and literacy development. *New Dir. Child Adolesc. Dev.* 2001:39–52. 10.1002/cd.14 11468865

[B91] Shalev R. S. Manor O. Kerem B. Ayali M. Badichi N. Friedlander Y. (2001). Developmental dyscalculia is a familial learning disability. *J. Learn. Disabil.* 34 59–65. 10.1177/002221940103400105 15497272

[B92] Silinskas G. Leppänen U. Aunola K. Parrila R. Nurmi J. E. (2010). Predictors of mothers’ and fathers’ teaching of reading and mathematics during kindergarten and Grade 1. *Learn. Instr.* 20 61–71. 10.1016/j.learninstruc.2009.01.002

[B93] Simmons F. R. Willis C. Adams A. M. (2012). Different components of working memory have different relationships with different mathematical skills. *J. Exp. Child Psychol.* 111 139–155. 10.1016/j.jecp.2011.08.011 22018889

[B94] Simon T. J. Hespos S. J. Rochat P. (1995). Do infants understand simple arithmetic? A replication of Wynn (1992). *Cogn. Dev.* 10 253–269. 10.1016/0885-2014(95)90011-X

[B95] Sirin S. R. (2005). Socio-economic status and academic achievement: a metaanalytic review of research. *Rev. Educ. Res.* 75 417–453.

[B96] Skwarchuk S. L. Sowinski C. LeFevre J. A. (2014). Formal and informal home learning activities in relation to children’s early numeracy and literacy skills: the development of a home numeracy model. *J. Exp.Child Psychol.* 121 63–84. 10.1016/j.jecp.2013.11.006 24462995

[B97] Slavin R. E. Lake C. Groff C. (2009). Effective programs in middle and high school mathematics: a best-evidence synthesis. *Rev. Educ. Res.* 79 839–911. 10.3102/0034654308330968

[B98] Smets K. Gebuis T. Defever E. Reynvoet B. (2014). Concurrent validity of approximate number sense tasks in adults and children. *Acta Psychol.* 150 120–128. 10.1016/j.actpsy.2014.05.001 24875582

[B99] Swanson H. L. Sachse-Lee C. (2001). Mathematical problem solving and working memory in children with learning disabilities: both executive and phonological processes are important. *J. Exp. Child Psychol.* 79 294–321. 10.1006/jecp.2000.2587 11394931

[B100] Tobia V. Bonifacci P. Marzocchi G. M. (2016). Concurrent and longitudinal predictors of calculation skills in preschoolers. *Eur. J. Psychol. Educ.* 31 155–174. 10.1007/s10212-015-0260-y

[B101] Tobia V. Bonifacci P. Marzocchi G. M. (2018). *SNUP - Senso del Numero: Prerequisiti.* Florence: Hogrefe editore.

[B102] Tomasetto C. Alparone F. R. Cadinu M. (2011). Girls’ math performance under stereotype threat: the moderating role of Mothers’ gender stereotypes. *Dev. Psychol.* 47 943–949. 10.1037/a0024047 21744956

[B103] Tosto M. G. Petrill S. A. Halberda J. Trzaskowski M. Tikhomirova T. N. Bogdanova O. Y. (2014). Why do we differ in number sense? Evidence from a genetically sensitive investigation. *Intelligence* 43 35–46. 10.1016/j.intell.2013.12.0071649.43.6.1497 24696527 PMC3969293

[B104] van Bergen E. Bishop D. van Zuijen T. de Jong P. F. (2015). How does parental reading influence children’s reading? A study of cognitive mediation. *Sci. Stud. Read.* 19 325–339. 10.1080/10888438.2015.1050103

[B105] van Bergen E. van der Leij A. de Jong P. F. (2014). The intergenerational multiple deficit model and the case of dyslexia. *Front. Hum. Neurosci.* 8:346. 10.3389/fnhum.2014.00346 24920944 PMC4041008

[B106] Van Herwegen J. Costa H. M. Passolunghi M. C. (2017). Improving approximate number sense abilities in preschoolers: PLUS games. *Sch. Psychol. Q.* 32 497–508. 10.1037/spq0000191 27936832

[B107] Von Aster M. G. Shalev R. S. (2007). Number development and developmental dyscalculia. *Dev. Med. Child Neurol.* 49 868–873. 10.1111/j.1469-8749.2007.00868.x 17979867

[B108] Westermann G. Mareschal D. Johnson M. H. Sirois S. Spratling M. W. Thomas M. S. (2007). Neuroconstructivism. *Dev. Sci.* 10 75–83. 10.1111/j.1467-7687.2007.00567.x 17181703

[B109] Xu F. Spelke E. S. (2000). Large number discrimination in 6-month-old infants. *Cognition* 74, B1–B11. 10.1016/S0010-0277(99)00066-9 10594312

[B110] Xu F. Spelke E. S. Goddard S. (2005). Number sense in human infants. *Dev. Sci.* 8 88–101. 10.1111/j.1467-7687.2005.00395.x 15647069

[B111] Zhang X. Lin D. (2015). Pathways to arithmetic: the role of visual-spatial and language skills in written arithmetic, arithmetic word problems, and nonsymbolic arithmetic. *Contemp. Educ. Psychol.* 41 188–197. 10.1016/j.cedpsych.2015.01.005

